# Fabrication of High-Performance Bamboo–Plastic Composites Reinforced by Natural Halloysite Nanotubes

**DOI:** 10.3390/molecules25092259

**Published:** 2020-05-11

**Authors:** Xiaobei Jin, Jingpeng Li, Rong Zhang, Zehui Jiang, Daochun Qin

**Affiliations:** 1 Department of Biomaterials, International Centre for Bamboo and Rattan, Beijing 100102, China; jinxiaobei@icbr.ac.cn (X.J.); zhangrong@icbr.ac.cn (R.Z.); 2 China National Bamboo Research Center, Chinese Academy of Forestry, Hangzhou 310012, China; lijp@caf.ac.cn

**Keywords:** bamboo-plastic composites, halloysite nanotubes, mechanical properties, thermal properties, water absorption

## Abstract

Bamboo-plastic composites (BPCs) as new biomass-plastic composites have recently attracted much attention. However, weak mechanical performance and high moisture absorption as well as low thermal stability greatly limit their industrial applications. In this context, different amounts of halloysite nanotubes (HNTs) were used as a natural reinforcing filler for BPCs. It was found that the thermal stability of BPCs increased with increasing HNT contents. The mechanical strength of BPCs was improved with the increase in HNT loading up to 4 wt% and then worsened, while the impact strengths were slightly reduced. Low HNT content (below 4 wt%) also improved the dynamic thermomechanical properties and reduced the water absorption of the BPCs. Morphological studies confirmed the improved interfacial compatibility of the BPC matrix with 4 wt% HNT loading, and high-concentration HNT loading (above 6 wt%) resulted in easy agglomeration. The results highlight that HNTs could be a feasible candidate as nanoreinforcements for the development of high-performance BPCs.

## 1. Introduction

Bamboo is considered an environmentally friendly material as a result of its high mechanical strength, renewability, biodegradability, and low cost in many countries [1]. As an extension of wood-plastic composites (WPCs), bamboo-plastic composites (BPCs) are a kind of biomass-plastic mix that consists of a variety of components, including bamboo fibers (BFs) and thermoplastic materials (e.g., polyethylene, polypropylene, polyvinylchloride, etc.) [2,3,4]. BPCs are intriguing due to their promising features with regard to low density, good thermal insulation, and good mechanical properties. They are also inexpensive, holding great potential for applications in decking, docks, landscaping timbers, fencing, etc. [5,6]. Additionally, the rational use of bamboo processing residues in BPCs could provide a new purpose for bamboo, easing the need for timber. This is of important practical significance [7].

However, bamboo fibers present a hydrophilic nature because of their cellulose and hemicellulose contents, causing high moisture absorption and poor interfacial bonding between fibers and thermoplastics. This property leads to reduced mechanical properties in composites [8,9]. To improve the mechanical properties of BPCs, BFs can be treated with a chemical modification agent (silanes, maleated polypropylene, etc.), which can improve the poor interfacial bonding between the thermoplastics and BFs [10,11]. The addition of nanofillers in polymer matrix is another way to enhance the overall properties of BPCs in a manner that is simpler and with a lower cost [12,13,14]. Because of their high aspect ratio, low density, and high Young’s modulus, nanofillers provide dramatic increases in the modulus, strength, heat resistance, and water absorption of composites [15,16,17].

Recent research has indicated that nanotechnology is a very promising field for improving the properties of BPCs [18]. Among various nanomaterials, halloysite nanotubes (HNTs) have been incorporated as a new type of green, unique, and promising reinforcing filler for thermosets and thermoplastic polymers [19,20,21]. HNTs (Al_2_Si_2_O_5_(OH)_4_·nH_2_O), a hydrated polymorph of 1:1 rolling phyllosilicate clay, are readily obtainable and biocompatible, providing great advantages compared with other traditional fillers such as carbon nanotubes [22,23]. HNTs can be readily dispersed in most polymer composites due to their rod-like geometry, low hydroxyl density on the surface, and lack of intertwining [24]. The characteristics of high aspect ratio, high surface area, and high mechanical strength make HNTs a good filler to promote the interaction of components and ultimately provide materials with high mechanical strength and thermal stability at low cost [25].

Low-density polyethylene (LDPE) is an important thermoplastic for bamboo/natural fiber–polymer composites [26,27]. However, its applications are limited due to its disadvantages such as low strength and modulus, low softening point, and so on [28]. So far, little work has been done on the incorporation of HNTs in bamboo fiber–LDPE blends. In this work, the feasibility of using HNTs to improve the properties of the bamboo fiber–LDPE composites was explored. Then, the mechanical, water absorption, and thermal properties of the BPCs were investigated.

## 2. Results and Discussion

### 2.1. Mechanical Properties

**Figure 1:**
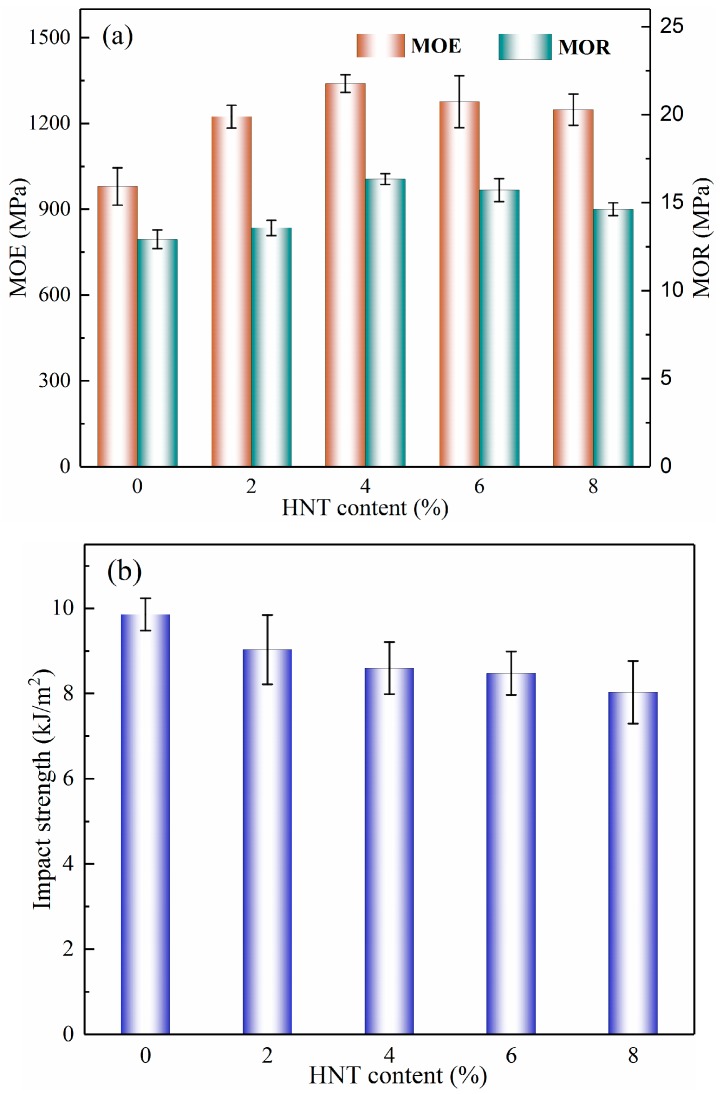
Mechanical properties of the bamboo–plastic composites (BPCs) with different contents of halloysite nanotubes (HNTs): (**a**) Flexural properties and (**b**) impact strength.

The effects of the HNT content on the flexural properties of BPCs are illustrated in Figure 1a. The modulus of rupture (MOR) and modulus of elasticity (MOE) of the BPCs were increased gradually as the HNT loading increased up to 4 wt%. Composites made with 4 wt% HNTs showed maximum strength and flexural modulus values that were increased by about 36.73% and 26.55%, respectively, compared with neat BPCs. However, the flexural properties of the BPCs moderately decreased as the HNT content increased from 6 wt% to 8 wt%. It is assumed that the decrease in mechanical properties was due to the agglomeration of nanoparticles as higher proportions of HNTs were loaded in the composites, as further supported by the SEM analysis.

Figure 1b shows that the impact strength of the composites decreased slightly as the HNT loading increased. The lowest impact strength was observed with the hybridization of 8 wt% HNTs, where the impact strength decreased by 18.52% compared with that of the control sample. The drop of strength at higher HNT loading was linked to an increased number of stress concentration points caused by the HNTs inside the system, as stress cracks required less energy to propagate through the matrix [29,30]. Similar results were reported by Khanjanzadeh et al. [31], who studied the properties of polypropylene-wood flour composites and found that the impact strength was decreased with increased nanoclay loading.

### 2.2. Interfacial Morphologies

**Figure 2:**
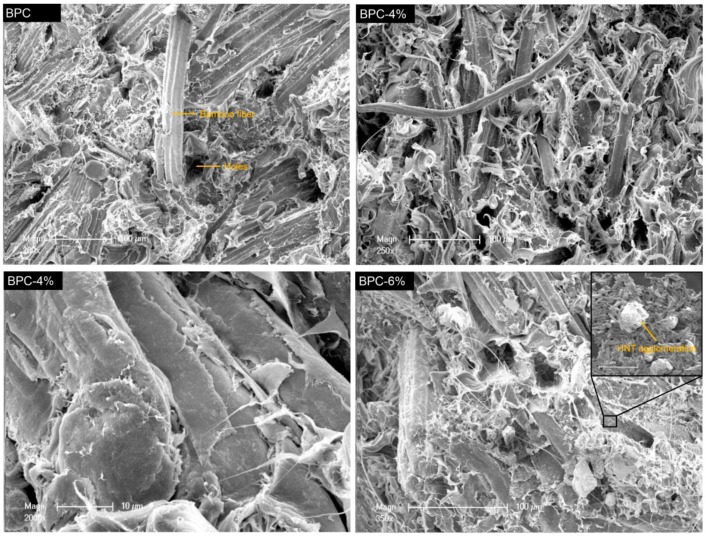
Scanning electron microscopy (SEM) images of impact fracture surfaces of the BPCs.

The morphological features of the fracture surfaces of BPCs were investigated with SEM (Figure 2). From the micrograph of BPC, it can be observed that bamboo fibers were pulled out of the matrix and micro holes appeared, suggesting a poor interfacial bonding between the BFs and LDPE matrix. Openings between the plastic and bamboo fibers were clearly visible, and could lead to higher water absorption and reduced mechanical properties. BPC-4% samples showed a fuzzy rupture interface with filamentous resin, and the fibers were surrounded by the polyethylene resin with well-dispersed HNTs. These nanotubes can form a “skeleton” in the polymers to increase the bonding at the fiber–matrix interface, enhancing the composite strength [32]. Wang et al. [14] also reported that inorganic nanoparticle impregnation treatments could improve the compatibility between bamboo fibers and polymer matrix, resulting in increased mechanical properties of the composites. As can be clearly seen from the fracture morphology of the BPC-6% sample, increasing the HNT loading to 6 wt% resulted in aggregation in the BPC matrix. The existence of HNT agglomerates is believed to reduce the mechanical properties of the composites. Tabari et al. [33] also found that there was no chemical interaction between the mineral nanofillers and other components in WPCs, and the aggregation of the fillers reduced the adherence of the matrix with the bamboo fiber.

### 2.3. Water Absorption

**Figure 3:**
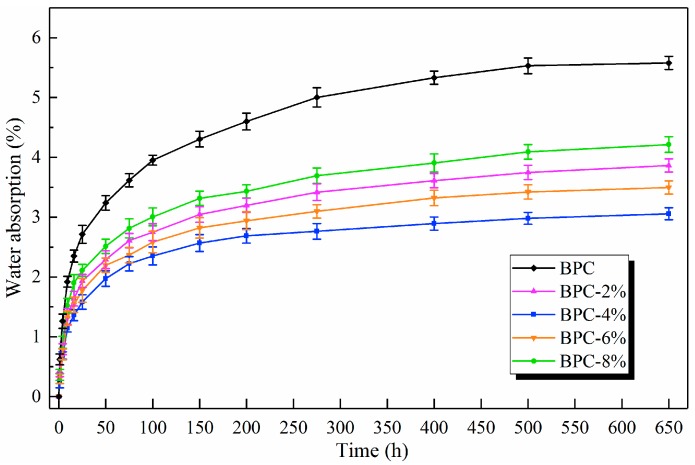
Water absorption curves of the BPCs.

Water absorption is one of the key parameters in the quality assessment of BPCs [34]. The results of the water absorption testing of the BPCs are shown in Figure 3. All composite samples absorbed water rapidly within initial stage of immersion process, with this subsequently and gradually slowing down until equilibrium. It is well known that neat LDPE does not absorb moisture because of its hydrophobic nature, the observed rise in water absorption capacity is attributed to the hydrophilic nature of bamboo fibers, as well as voids and micro-gaps between the fiber and matrix. Despite the hydrophilicity of the HNT fillers, increasing the HNT loading to the system resulted in a reduction in the uptake of water, and the BPC-4% sample showed the lowest water absorption (3.04%). This may have been because the presence of HNT fillers in the composite system resulted in a denser nanocomposite network, which then interfered with the passage of water molecules into the composite [23]. Alhuthali et al. [35] also reported that the amount of water absorbed decreased as the HNT loading increased in composites, thus achieving the desirable effect of HNTs in reducing water absorption. With higher proportions of HNTs (6 wt% to 8 wt%), the agglomeration of nanoparticles was responsible for higher water uptake values, as confirmed by the SEM micrographs.

### 2.4. Thermogravimetric Analysis

**Figure 4:**
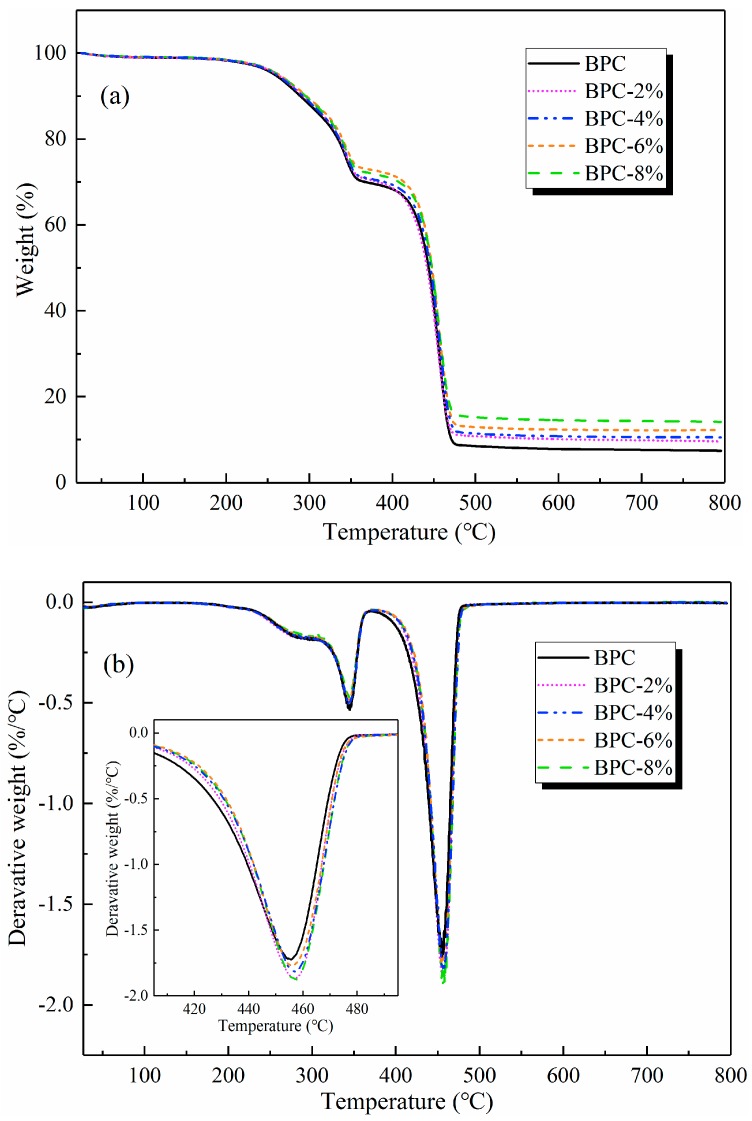
Thermogravimetric analysis of the BPCs: (**a**) TGA and (**b**) DTG.

Figure 4a demonstrates the thermogravimetric analysis (TGA) results of the BPCs containing various amounts of HNTs, and the data are summarized in Table 1. It can be observed that the thermal degradation of HNT-filled samples occurred in two-step degradation process. The first step that occurred from approximately 200 to 400 °C was mainly due to the decomposition of BFs. The second stage in the TGA that appeared above 400 °C was associated with the thermal degradation of the LDPE backbone, the dehydroxylation of structural alumina groups present in HNTs, and the further thermal oxidative degradation of the char residue [36,37]. The incorporation of 4 wt% HNTs increased the initial decomposition temperatures (*T*_i_) by 6.42 °C and 17.61 °C for the first and second steps, respectively, compared with the control sample. The residual weight of BPCs at 800 °C and the temperatures at different weights (80%, 60%, 40%, and 20%) were increased with an increase in HNT loading in composites.

From the differential thermogravimetric (DTG) curves (Figure 4b), it was found that the addition of HNTs slightly shifted the peak degradation temperatures of the BPCs towards higher temperatures. The temperature of the maximum mass loss rate was found at 459.67 °C for BPC-4%, which was higher than that of the control sample (451.56 °C). Based on all the results above, it is believed that the thermal resistance of HNTs themselves is responsible for the improved thermal stability of BPCs. In fact, the hollow structure of HNTs is expected to entrap decomposition products, which could play a positive role in forming of a char layer and delaying the mass transfer [24]. Additionally, the improvement in thermal stability is also attributable to the released water from the degradation of interlayer aluminum oxide in HNTs and the metal oxides in contamination of HNTs efficiently trapping free radicals during the degradation reaction [38].

### 2.5. Dynamic Mechanical Analysis

**Figure 5:**
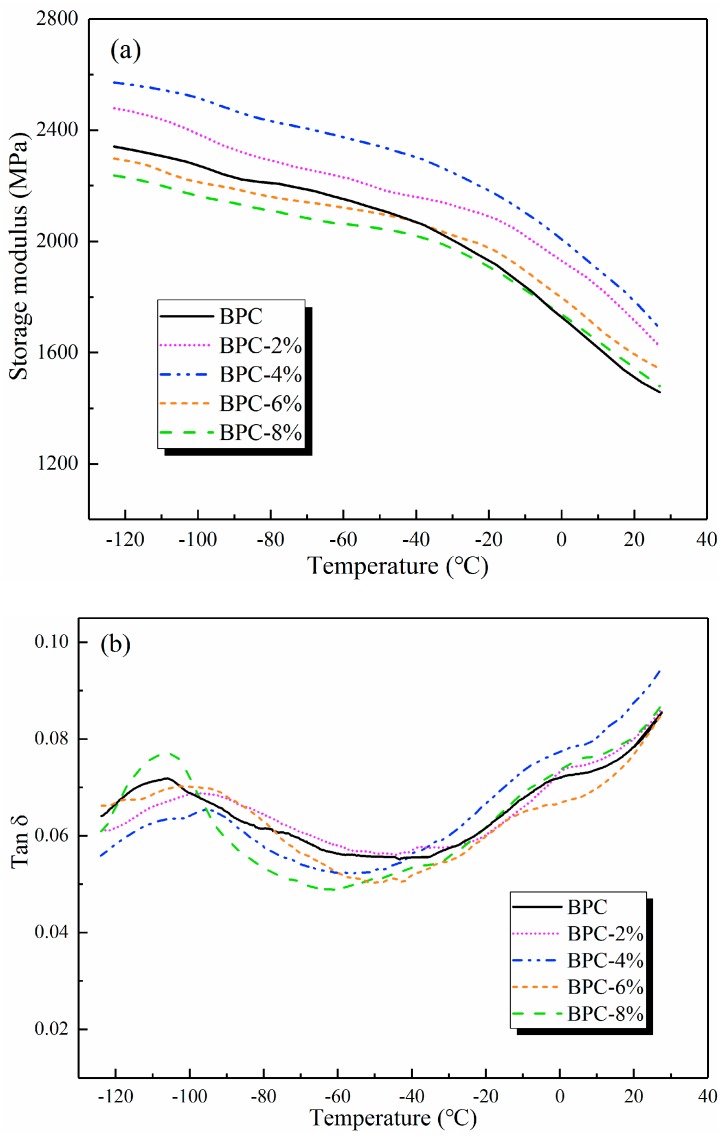
Plots of (**a**) storage modulus and (**b**) tan δ of the BPCs.

Figure 5a shows the variation of storage modulus with temperature for the BPCs. It can be seen that the storage modulus of the BPCs increased considerably with higher HNT loading up to 4 wt% at the same temperature. This was ascribed to the restriction of the chain mobility in the crystalline phase [39]. However, the storage modulus values of the BPCs above 6 wt% HNT loading were lower than those of the neat BPCs. It can be noted that excess nanoparticle loading may decrease the crystallinity of the matrix due to the presence of imperfect crystals nucleated by the HNT particles [40]. In addition, the voids (Figure 2) and the nanoparticle agglomeration during sample preparation may weaken the storage modulus.

The curves for the mechanical tan δ of the BPCs against the temperature are shown in Figure 5b. The temperature corresponding to the maximum in tan δ (between −120 and −90 °C) was taken as the glass-transition temperature (*T*_g_) of the composites. It can be seen that with increasing content of HNTs up to 6 wt%, the *T*_g_ values of the composites were increased and a narrower hump was observed, indicating a constrained mobility of the chains of the polymeric matrix [41]. The composites with 2 wt%, 4 wt%, and 6 wt% HNT loading showed lower peak heights of tan δ values than the neat BPCs. Such observations could be attributed to lower viscoelastic energy dissipation in the glass-transition region of HNT-filled composites than in the neat composites [42]. Furthermore, the BPCs with 8 wt% HNT loading exhibited a higher value of *T*_g_ compared with those of the neat composites. This can probably be attributed to the poor interactions between filler and polymer and the imperfect crystals nucleated by the agglomeration of HNT particles [40].

## 3. Materials and Methods

### 3.1. Materials

BFs from Moso Bamboo (*Phyllostachys edulis*) (100 mesh size) were purchased from Wood River Weihua Spices Factory (Jiangmen, China). The LDPE (density = 0.95 g/cm^3^ and melt flow index = 2.0 g/10 min) was purchased from Suzhou Joe Shun Plastic Co., Ltd. (Suzhou, China). The HNTs, which were obtained from Danjiangkou Mineral Factory (Hubei, China), were in a tubular form with an external diameter typically smaller than 100 nm, internal diameter of 20 nm, and length of 0.2 μm to 2 μm. Vinyl triethoxysilane (NanJing YouPu Chemical Company, Nanjing, China) was used as the coupling agent. Ethanol and acetic acid were purchased from Beijing Chemical Company (Beijing, China). All of the chemicals from commercial sources were of reagent grade and were used without further purification.

### 3.2. Preparation of BPC Samples

The BFs was dried at 103 °C for 8 h to remove all the moisture and then treated with vinyl triethoxysilane according to the following procedure [43]. The coupling agent solution was prepared at a concentration of 4 vol% with ethanol. The solution was heated to 60 °C and stirred for 10 min after pH adjustment to the range 4.5 to 5.5 using acetic acid. The equal mass of the solution based on BFs was sprayed onto the surface of the BFs uniformly at room temperature. The treated BFs was placed in a ventilated place for evaporating the alcohol and then dried in an oven at 100 °C for 24 h to remove the remaining moisture.

**Figure 6:**
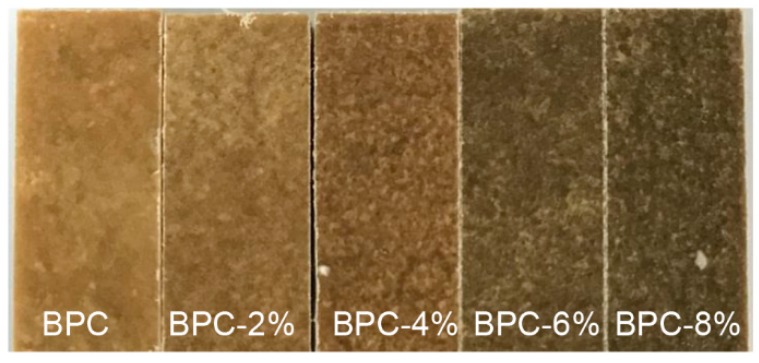
Images of the BPC samples made with different contents of HNTs.

The BFs and LDPE, with a weight ratio of 40%:60% (*w*/*w*), were premixed for 60 min at 50 °C in a malaxator (NH-1, Guancheng Machine Company, Jiangsu, China). The HNT nanoparticles were added slowly to the mixtures at concentrations of 2 wt%, 4 wt%, 6 wt%, and 8 wt% (based on the total matrix) while the mixing process occurred. Before hot pressing, the mixtures were weighed accurately to ensure the target density of the BPCs was 1.0 kg/m^3^. The mixtures were subsequently placed in a mold with the dimensions 250 mm × 90 mm × 3.5 mm and hot-pressed at 150 °C for 30 min at a pressure of 6 MPa (3895, Carver Co., Ltd., Wabash, IN, USA). Finally, the composites were removed when the temperature decreased below 35 °C. Control samples without any fillers were also prepared. The BPCs with 2 wt%, 4 wt%, 6 wt%, and 8 wt% HNTs were denominated BPC-2%, BPC-4%, BPC-6%, and BPC-8%, respectively. The final BPCs are shown in Figure 6.

### 3.3. Characterization of the BPCs

The flexural properties were measured with three-point static bending tests in accordance with ASTM D790 (2010) using a universal mechanical tester (Instron 5582, Norwood, MA, USA). The dimensions (length × width × thickness) of the samples were 90 mm × 15 mm × 3.5 mm, and the loading speed was 0.2 mm/min.

Impact tests were conducted in accordance with ASTM D6110 (2010) using an impact-type testing machine (XJJ–5, Kecheng Testing Machine Company, Chengdu, China). The dimensions of the impact test samples were the same as those of the flexural test samples. At least five duplicate samples were used for each test.

Scanning electron microscopy (SEM) (FEG-XL30, FEI Company, Hillsboro, OR, USA) was used to determine the surface morphology of the BPC cross sections subjected to impact testing. The samples were first sputtered with a layer of gold and then observed using at an accelerating voltage of 10.0 kV.

Water absorption was performed in accordance with ASTM D570–98 (2005). Before testing, the weight of each specimen was measured with a precision of 0.001 g. Conditioned samples were soaked in distilled water and kept at room temperature for 650 h. The specimens were periodically taken out of the water, wiped of surface water, and then reweighed and immediately put back into the water. The water absorption (*W_A_*, %), calculated from the average of five samples, was determined as:

*W_A_* (%) = (*M_t_* − *M_0_*)/*M_0_* × 100

where *M_t_* is the mass (g) of the sample at time *t* (h) after immersion and *M_0_* is the mass (g) of the sample before immersion.

Thermogravimetric analysis (TGA) was performed with a TGA instrument (TA Q 500, TA Instruments, New Castle, PA, USA). Samples that weighed between 6 mg and 10 mg were heated from room temperature to 800 °C at a heating rate of 10 °C /min under a nitrogen atmosphere with a flow rate of 40 mL/min.

The dynamic mechanical properties (DMA) of BPCs were investigated according to the ASTM D5418–01 test in the dual cantilever mode with dynamic mechanical analysis (Q800, TA Instruments). Samples with the dimension of 64 mm × 15 mm × 3.5 mm were tested at a heating rate of 2 °C/min and a frequency of 1 Hz. The storage modulus and loss tangent (tan δ) were recorded over a temperature range from −130 °C to 40 °C.

## 4. Conclusions

In summary, halloysite nanotubes (HNTs) are excellent reinforcing nanomaterials for BPCs due to their high mechanical strength, thermal stability, and abundance. We established HNTs as a natural nanofiller in BPC matrices. The incorporation of HNTs (below 4 wt% loading) enabled BPCs to achieve higher flexural properties, thermal stability, viscoelastic properties, and low water absorption due to the uniform dispersion of HNTs imparting better interfacial bonding between the bamboo fiber and the matrix polymer. It is important to emphasize that excessive HNT loading (above 6 wt%) resulted in an agglomeration of nanoparticles, which was evidenced by SEM analysis, and this caused the reduction in properties of the composites. It is concluded that HNTs in appropriate proportions could be employed as a promising nanofiller for developing high-performance BPCs.

## Figures and Tables

**Table 1:** Thermogravimetric analysis (TGA) results of the BPC samples.

Sample	*T*_i_ (°C) 1 Step	*T*_i_ (°C) 2 Step	Temperature (°C) at Different Weights				Residual Weight (%) at 800 °C
			80%	60%	40%	20%	
BPC	222.02	389.01	331.74	430.73	450.30	460.67	7.41
BPC-2%	226.88	395.59	333.98	432.52	451.26	463.51	9.58
BPC-4%	228.44	406.62	335.01	435.23	452.72	464.33	10.53
BPC-6%	231.52	409.14	337.87	437.48	453.55	465.54	12.26
BPC-8%	238.91	411.06	338.66	438.09	453.76	466.79	14.12

*T*_i_: initial decomposition temperature.
